# Antimicrobial Resistance Among Uropathogens: Surveillance Report From South India

**DOI:** 10.7759/cureus.12913

**Published:** 2021-01-26

**Authors:** Uma Ravishankar, Sathyamurthy P., Premamalini Thayanidhi

**Affiliations:** 1 Internal Medicine, Sri Ramachandra Institute of Higher Education and Research, Chennai, IND; 2 Microbiology, Sri Ramachandra Institute of Higher Education and Research, Chennai, IND

**Keywords:** amr surveillance, uropathogens, uti

## Abstract

Background

Urinary Tract Infection (UTI) is one of the most common infections encountered in clinical practice. Evidence supports that empirical treatment guidelines based on local bacterial spectrum and antimicrobial resistance (AMR) surveillance provide the best clinical results and also prevent the emergence of resistant strains. Antimicrobial resistance has been increasing at an alarming rate throughout the world. This warrants continuous reporting and surveillance of the emergence of AMR among the uropathogens across regions and nations.

Materials and methods

A retrospective cross-sectional study using antibiograms of adult patients with culture-proven UTI during January 2011 and January 2017 was done. Comparative analysis was performed between the two study periods for the prevalence, changing trends of antimicrobial resistance, and usage of antimicrobials for testing.

Results

The commonest organism cultured during each study period was *Escherichia coli *(56.6% and 51.6%). The most frequently tested antibiotics were ampicillin (97%, 88%), amikacin (85%, 85%), nitrofurantoin (95%, 95%), cephalexin (84%, 93%), and norfloxacin (83%, 83%). There was a significant increase in resistance proportion noted for imipenem (by 29.8%), meropenem (by 18.3%), ertapenem (by 24.9%), ciprofloxacin (by 26.5%), nitrofurantoin (by 11.2%), amikacin (by 8.7%), and cefotaxime (by 7.4%) in 2017 as compared to 2011. A significant increase in susceptibility was seen for tobramycin (by 32.5%), cefepime (by 14.4%), and polymyxin (by 12.6%) in 2017 when compared to 2011.

Conclusion

Our analysis has shown that there is an alarmingly increasing trend for AMR among uropathogens in this region as compared to developed countries. Data on changing trends of antimicrobial testing and reporting might help in strengthening antimicrobial surveillance.

## Introduction

Urinary Tract Infection (UTI) is one of the most common infections encountered in clinical practice. Empirical treatment for both complicated and uncomplicated UTI has been practiced throughout the world because a failure in timely treatment might lead to increased morbidity and mortality [1,2,3]. Empirical treatment could be a failure if the local resistance rates of the uropathogens for the used antibiotic exceed 20 % [1,4]. But this has to be balanced with the inappropriate usage of broad-spectrum antibiotics leading to the emergence of resistant strains [4]. In fact, unregulated and extensive antibiotic usage has been shown to be the principal reason for the development of antimicrobial resistance (AMR) [5,6]. Usage of the correct drug in the correct dosage for the shortest clinically effective period has been shown to be the basis for antimicrobial stewardship (AMS) in urological infections [7]. It is well known that culture reports form the basis for treating physician’s intention in changing the broad-spectrum empirical antibiotic to one which is more specific and with a narrow spectrum. The antimicrobial testing and reporting pattern worldwide have been largely based on recommended guidelines from agents like CLSI (Clinical and Laboratory Standards Institute) and EUCAST (The European Committee on Antimicrobial Susceptibility Testing) who in turn depend upon the global antimicrobial susceptibility data for forming testing guidelines.

Mathematically, an inappropriate increase or decrease in testing and reporting rates for antibiotics might cause an erroneous estimation of prevalence in resistance rates. This factor has not been considered in antimicrobial surveillance protocols to a large extent till now. Evidence supports that empirical treatment guidelines based on local bacterial spectrum and AMR surveillance provide the best clinical results and also prevent the emergence of resistant strains [8]. Thus, AMR surveillance forms the link between testing and treatment guidelines, which are often provided by different kinds of agencies. It is very much evident that AMR has been increasing at an alarming rate throughout the world with the appearance of more extended-spectrum beta-lactamase (ESBL)infections [5]. World Health Organization's global plan of action on AMR has stressed AMR surveillance across nations as an important strategy for reducing AMR [9]. Also, it has been shown that the emergence of AMR is proportionately more in the low- and middle-income countries [8,10]. This warrants continuous reporting and surveillance of the emergence of AMR among the uropathogens across regions and nations. This study aims at reporting the prevalence, trend, and rate of emergence of AMR among the uropathogens from South India by doing a comparison between 2011 and 2017. We also aim to describe the pattern and trend in the usage of antimicrobials for testing and reporting urine cultures during the two study periods.

## Materials and methods

A retrospective cross-sectional study using antibiograms of patients who presented to our institution with culture-proven UTI during January 2011 and January 2017 was done. Culture and sensitivity reports of urine samples collected from both hospitalized and non-hospitalized adult (age ≥18 years) patients were included in the study. Culture reports of pediatric age group and ones with insignificant colony counts(<10^5 ^CFU/ml) were excluded from the study. Semi-quantitative culture of urine specimens on CLED (cystine lactose electrolyte deficient) media was used for colony counting. Significant bacteriuria is defined as voided urine sample containing more than 10^5^ CFU/ml of urine in pure culture using a standard calibrated bacteriological loop. Pure growth of a single bacterial species with a colony count of >10^5^ CFU/ml after overnight incubation was considered as indicative of infection. Identification of the bacteria was done by routine biochemical reactions. Antibiotic sensitivity testing was done by the disk diffusion method in Muller-Hinton agar according to the prevailing CLSI guidelines.

The calculated sample size was a minimum of 238 samples for each study period with an absolute precision of 5%(0.05), and an estimated prevalence rate of 19% (0.19) based on an earlier study from this region [11]. The sample size was calculated using the following formula:

$n=Z^2 \times \frac{P(1-P)}{d^{2}}$

Where* n* is the sample size, *Z* is the statistic corresponding to the level of confidence (1.96), *P* is the expected prevalence, and *d* is precision (corresponding to effect size). For maintaining similarity, all eligible samples collected during the similar period (month of January) of 2011 and 2017 were included.

Data were extracted from hospital records manually. They were entered into Microsoft Excel (Microsoft Corporation, Redmond, WA, USA) and analyzed using the Statistical Package for the Social Services (SPSS) software version 26 (IBM Corp., Armonk, NY) at Chennai, India. Distributive statistics of the bacterial spectrum, antimicrobial testing, and antimicrobial resistance were represented in percentage (%) using tables. Pie charts were used to express the distribution of uropathogens during the two study periods. Trends of increase and decrease in the degree of resistance were represented by bar diagrams. Comparative analysis was performed between the two study periods for the prevalence of antimicrobial resistance. Categorical variables were compared using Pearson’s Chi-square test. An alpha value of 0.05 was considered for statistical significance. The study was approved by the institutional ethics committee.

## Results

Antibiograms of 265 urine culture samples in the month of January 2011 and 248 urine culture samples in the month of January 2017 (total 513) were analyzed in the study.

The most common organism cultured during both study periods was *Escherichia coli *(56.6% and 51.6%). The other common organisms were *Klebsiella pneumoniae *(14.7%, 16.5%) and *Enterococcus faecalis *(11.6%, 12%). The prevalence of the other organisms was less than 10% in both study periods. In 2017, *Providencia sp.* (2.4%), *Morganella morganii *(2.8%), Coagulase-negative Staphylococci (CoNS) (0.4%), and *Klebsiella oxytoca *(0.8%) were seen in the causative spectrum, while they were not seen during 2011 (Table 1).

**Table 1:** Spectrum of bacterial uropathogens isolated during the study periods.

Microorganism	2011 (n=265, 100%)	2017 (n=248, 100%)
Escherichia coli (E. coli)	150(56.6)	128(51.6)
Klebsiella pneumoniae (Kleb pneumon)	39(14.7)	41(16.5)
Enterococcus faecalis (E. faecalis)	31(11.6)	30(12)
Pseudomonas aeruginosa (Pseudomonas)	12(4.5)	13(5.2)
Acinetobacter species	11(4.1)	7(2.8)
Proteus mirabilis	5(1.8)	6(2.4)
Staphylococcus aureus (Staph aureus)	6(2.2)	3(1.2)
Citrobacter freundii (Citrobacter)	4(1.5)	3(1.2)
Streptococcus species	2(0.7)	1(0.4)
Proteus vulgaris	5(1.8)	0(0)
Providencia species	0(0)	6(2.4)
Morganella morganii	0(0)	7(2.8)
Coagulase-negative Staphylococcus (CoNS)	0(0)	19(0.4)
Klebsiella oxytoca (Kleb oxytoca)	0(0)	2(0.8)

The most frequently tested antibiotics were ampicillin (97%, 88%), amikacin (85%, 85%), nitrofurantoin (95%, 95%), cephalexin (84%, 93%), and norfloxacin (83%, 83%). There was a significant increase in testing for piperacillin-tazobactam (25% vs 83%), cefoperazone sulbactam (38% vs 83%), cefotaxime (29% vs 92%), cefepime (9% vs 35%), ciprofloxacin (3% vs 15%), levofloxacin (2% vs 36%), and tobramycin (16% vs 32%) during 2017 when compared to 2011. Fosfomycin was present in the testing panel during 2017 and was not there during 2011. There was a significant reduction in testing frequency for imipenem during 2017 as compared to 2011 (84% vs 33%) (Table 2).

**Table 2:** Usage of various antibiotics for sensitivity testing during the study periods

Antibiotic	Percentage tested for in 2011 (n=265, 100%)	Percentage tested for in 2017 (n=248, 100%)
Ampicillin	97	88
Cephalexin	84	93
Cefuroxime	60	57
Cefotaxime	29	95
Ceftazidime	29	23
Cefepime	9	35
Cefoperazone sulbactam	38	83
Piperacillin tazobactam	25	83
Imipenem	84	33
Meropenem	23	33
Ertapenem	18	21
Cotrimoxazole	80	83
Nitrofurantoin	95	95
Norfloxacin	83	83
Ciprofloxacin	3	15
Levofloxacin	2	36
Gentamycin	12	14
Amikacin	85	85
Tobramycin	16	32
Fosfomycin	0	13
Polymixin	15	28
Vancomycin	12	10
Linezolid	12	10

**Figure 1:**
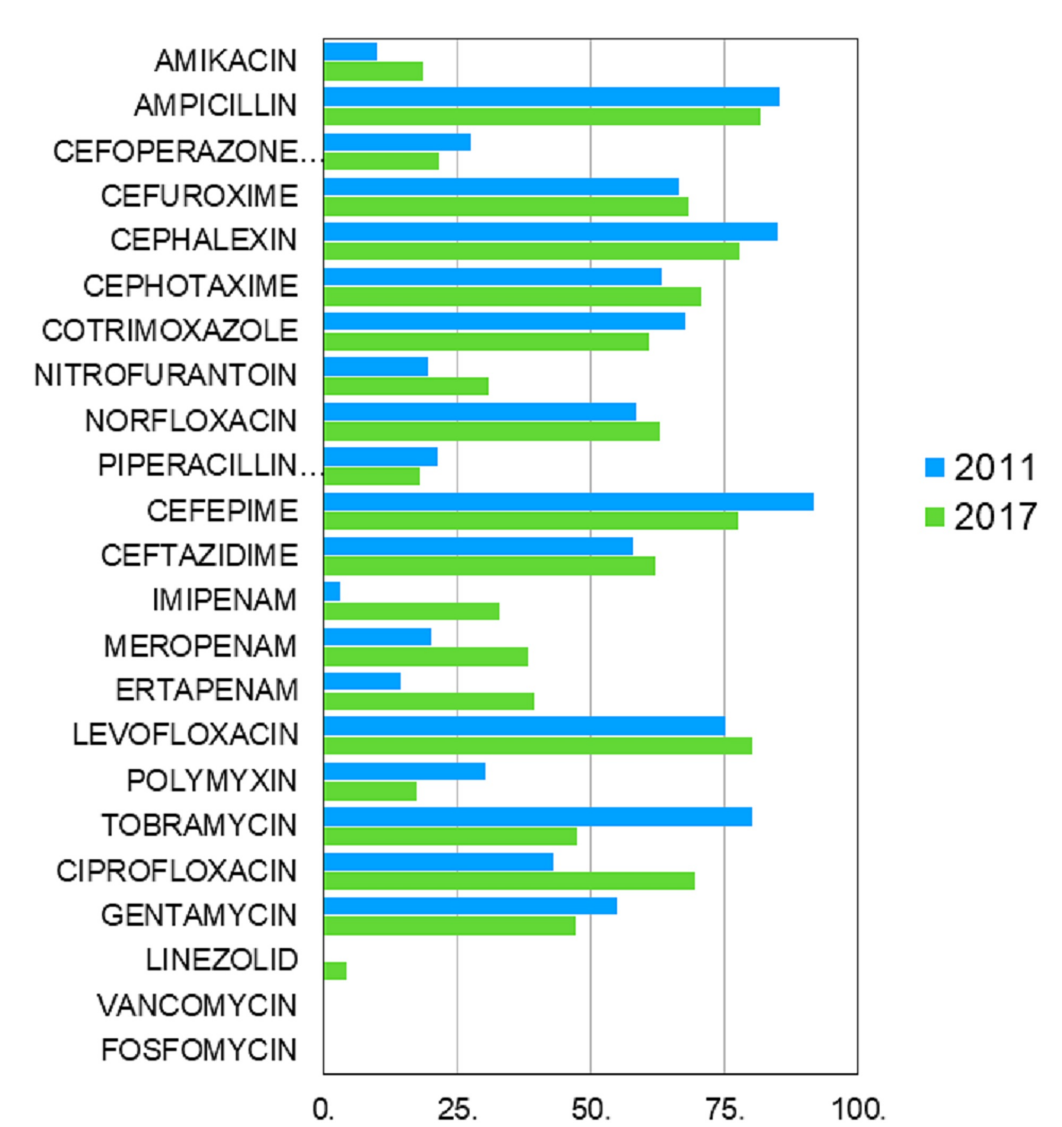
Changing trends in the prevalence of resistance (%) of various antibiotics over six years

There was a significant increase in resistance proportion noted for imipenem (29.8%), meropenem (18.3%), ertapenem (24.9%), ciprofloxacin (26.5%), nitrofurantoin (11.2%), amikacin (8.7%), and cefotaxime (7.4%) in 2017 as compared to 2011 (Table 3, Figure 1). There was a marginal increase in resistance percentage for levofloxacin (5%), linezolid (4.2%), norfloxacin (4.4%), ceftazidime (4.2%), and cefuroxime (1.6%) in 2017 as compared to 2011 (Table 3, Figure 1). Among these the increase in resistance for amikacin (p=0.006), nitrofurantoin (p=0.003), imipenem (p=0.000), meropenem (p=0.015), and ertapenem (p=0.004) achieved statistical significance (Table 3).

**Table 3:** Prevalence of resistance of various antibiotics among uropathogens compared between the two study periods * number of resistant isolates; ^#^ total number of isolates tested

Antimicrobial	2011 -R*	2011-total^#^	%	2017-R*	2017-total^#^	%	P-value
Ampicillin	218	256	85.2	179	219	81.7	0.190
Cephalexin	189	223	84.8	180	232	77.6	0.033
Cefuroxime	107	161	66.5	95	141	68.1	0.43
Cefotaxime	48	76	63.2	166	235	70.6	0.14
Ceftazidime	44	76	57.9	36	58	62.1	0.379
Cefepime	22	24	91.7	68	88	77.3	0.095
Cefoperazone sulbactam	28	102	27.5	44	206	21.4	0.148
Piperacillin-tazobactam	14	66	21.2	37	205	18	0.342
Imipenem	7	223	3.1	27	82	32.9	0.000
Meropenem	12	60	20	31	81	38.3	0.015
Ertapenem	7	49	14.3	20	51	39.2	0.004
Cotrimoxazole	144	213	67.6	125	206	60.7	0.084
Nitrofurantoin	49	252	19.4	72	235	30.6	0.003
Norfloxacin	129	221	58.4	130	207	62.8	0.201
Ciprofloxacin	3	7	42.9	25	36	69.4	0.179
Levofloxacin	3	4	75	72	90	80	0.601
Gentamycin	17	31	54.8	17	36	47.2	0.353
Amikacin	22	225	9.8	39	211	18.5	0.006
Tobramycin	36	45	80	38	80	47.5	0.000
Fosfomycin	0	0	0	0	33	0	
Polymixin	12	40	30	12	69	17.4	0.099
Vancomycin	0	33	0	0	24	0	
Linezolid	0	33	0	1	24	4.2	0.0421

**Figure 2:**
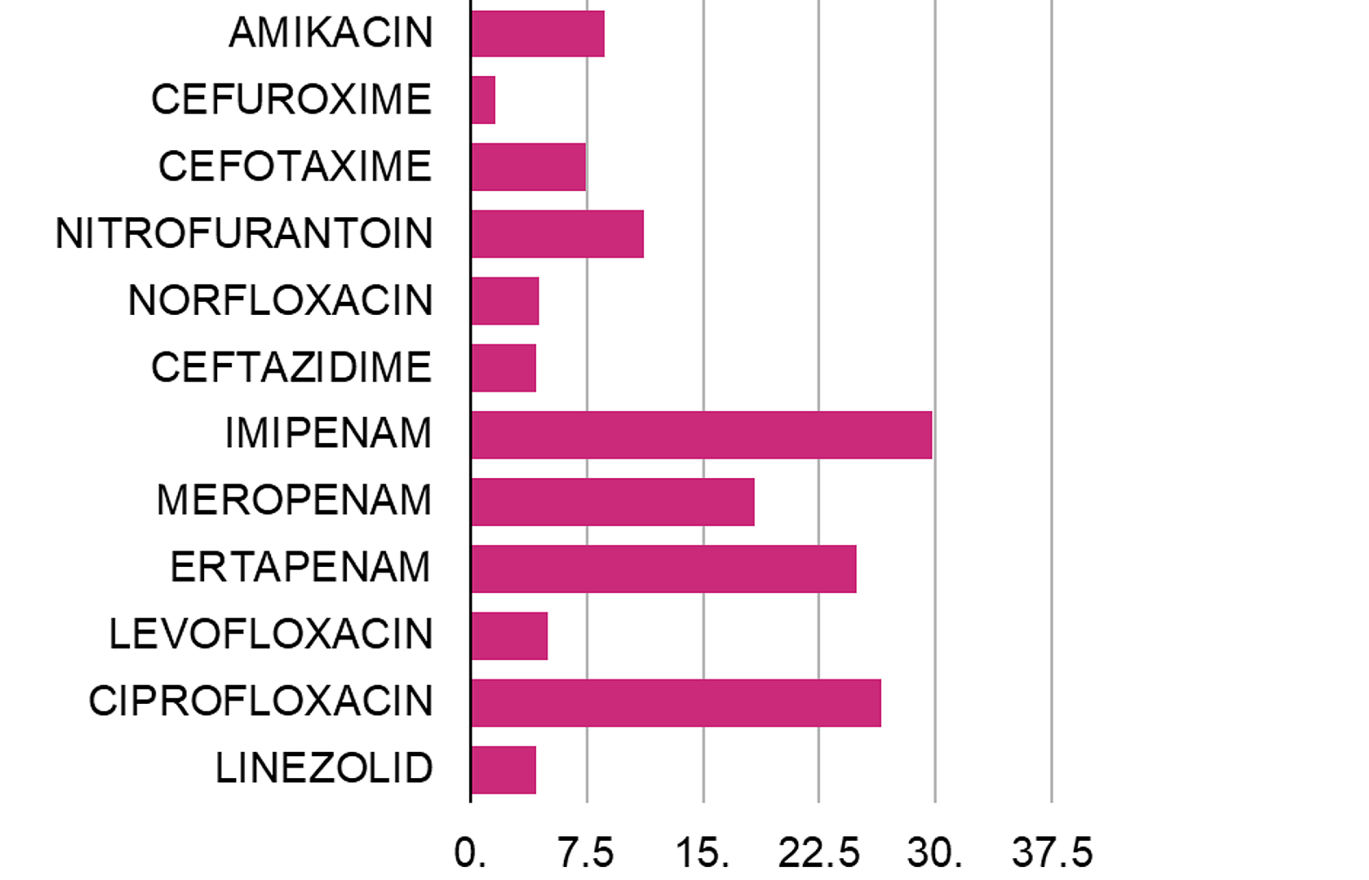
Increase in resistance (%) for antimicrobials observed during 2017 compared to 2011

A significant increase in susceptibility was seen for tobramycin (32.5%), cefepime (14.4%), and polymyxin (12.6%) (Table 3, Figure 2). There was a marginal decrease in resistance for ampicillin (3.5%), gentamycin (7.6%), cotrimoxazole (6.9%), cephalexin (7.2%), cefoperazone sulbactam (6.1%), and piperacillin-tazobactam (3.2%) (Table 3, Figure 2). The decrease in resistance for tobramycin achieved statistical significance in the comparison (p=0.000) (Table 3). Vancomycin susceptibility was 100 percent during both study periods. Fosfomycin susceptibility for tested isolates was 100% in 2017 (Table 3).

## Discussion

The spectrum of uropathogens causing both hospital-acquired urinary tract infection (HAUTI) and community-acquired urinary tract infection (CAUTI) has been more or less similar and constant over periods throughout the world. In our analysis, in both periods, the most common organisms were *Escherichia coli *(56.6% vs 51.6%), *Klebsiella pneumoniae *(14.7% vs 16.5%), *Enterococcus faecalis *(11.6% vs 12%), and *Pseudomonas aeruginosa *(4.5% vs 5%). This spectrum is similar to the previous studies by Meena et al. [12], and Shah et al. [6] from India; Sorlozano et al. [13] from Spain; Eure et al. [14] and Sharma et al. [15] from Grenada; and Lagunas-Rangel [16] from Mexico. Most authors have reported *Escherichia coli *and *Klebsiella pneumoniae *among the top three causative organisms for UTI (Table 4), which is similar to our study. The causative spectrum is very much similar, with the top six organisms having more or less the same prevalence in both the periods of our study (Table 1). This aids in doing a fair comparison between the two study groups.

**Table 4:** Spectrum of uropathogens reported by various authors from different regions. E. coli: *Escherichia coli*; E. faecalis: *Enterococcus faecalis*; Staph aureus: *Staphylococcus aureus*;* *others: other uropathogens; sps.: species % approximated to the nearest whole numbers

Taniece R. Eure et al. [14] United States - 2020	Antonio Sorlozano et al. [13] Spain - 2014	Francisco Alejandro Lagunas-Rangel et al. [16] Mexico - 2018	Mahadev Meena et al. [12] India -2018	A. Muhammad et al. [17] Pakistan - 2020	Kald BeshirTuem et al. [18] Ethiopia - 2017	Deepak Sharma et al. [15] Grenada - 2019	Seyed Abdol Reza Mortazavi-Tabatabaei et al. [19] Iran - 2019	Latika J Shah, et al. [6] Western India - 2015	Our study (2017 data)
E. coli (41%)	E. coli (55%)	E. coli (68%)	E. coli (67%)	E. coli (68%)	E. coli (48%)	E. coli (37%)	E. coli (62%)	E. coli (61%),	E. coli (52%)
Proteus sps. (14%)	E.faecalis (18%)	E. faecalis (3%)	Klebsiella sps. (18%)	Klebsiella sps. (9%)	Klebsiella sps. (16%)	Group b enterococcus (31%)	Klebsiella sps. (13%)	Kelbsiella sps. (14%)	Klebsiella sps. (17%)
Klebsiella sps. (13%)	Klebsiella sps. (10%)	Klebsiella sps. (2%)	E. faecalis (6%)	Staph aureus (5%)	Pseudomonas aeruginosa (7%)	Klebsiella sps. (11%)	Staph aureus (12%)	Acinetobacter sps.(10%),	E. faecalis(12%)
Others (32%)	Others (17%)	Others (27%)	Others (9%)	Others (18%)	Others (29%)	Others (21%)	Others (13%)	Others (16%)	Others (19%)

Testing and reporting for various antimicrobials

In our institution, the antimicrobial susceptibility testing and reporting have been based on CLSI guidelines. About 88% of the reporting in 2011 and 86% of the reporting in 2017 were for the three groups of pathogens namely Enterobacteriaceae, Enterococcus sp., and Pseudomonas sp. Hence, the pattern of antimicrobial susceptibility testing and reporting would be largely influenced by these organisms during the study periods.

In comparison with 2011, there has been a significant increase in testing and reporting for piperacillin-tazobactam, cefoperazone sulbactam, cefotaxime, cefepime, ciprofloxacin, and levofloxacin in 2017 (Table 2). All these agents were in the selective reporting group B of CLSI [20,21] guidelines during both the periods, except for piperacillin-tazobactam, which has been moved to routine reporting group A for pseudomonas from selective reporting group B in 2017 [21]. Increased resistance rates of amikacin, quinolones seen in 2017 [21] could be the reason for increased testing and reporting of cefotaxime, cefepime, cefoperazone sulbactam, and piperacillin-tazobactam. Another contributing factor could be the inclusion of piperacillin-tazobactam in routine reporting group A for Pseudomonas sp. in the 2017 CLSI guidelines [21]. The testing and reporting for imipenem showed a significant reduction in 2017 as compared to 2011 (33% vs 84%) (Table 2). This decrease for imipenem is perhaps explained by the increase in susceptibility observed for tobramycin, which is in routine reporting group A [20,21] for both Enterobacteriaceae, which leads to reduced testing for selective antibiotics (imipenem) group B in CLSI guidelines 2017 [21]. Also, in the 2017 CLSI guidelines, fosfomycin has been included for the first time in the supplemental group U testing for urinary isolates of *E. coli*. Fosfomycin showed 100% sensitivity for the isolates. This again could have contributed to the reduction in testing and reporting for the selective antibiotic imipenem. The analysis shows that the frequency of testing for the selective and broad-spectrum antibiotics is influenced by the pattern and prevalence of antimicrobial resistance among the causative organisms and the testing guidelines followed. The reported sensitivity pattern in turn shall be incorporated in treatment guidelines. This underlines the importance of strict monitoring at various levels, including the laboratory, for preventing the emergence of AMR. More longitudinal studies might be required to understand the influence of local AMR on the frequency of testing and reporting for antimicrobials, which would influence the antibiotic prescription and future treatment guidelines.

Prevalence of antimicrobial resistance

Antimicrobial resistance has been reported worldwide, but the prevalence has been varying across geographical regions [22] and study settings. Study for Monitoring Antimicrobial Resistance Trends (SMART) data had found that the prevalence of ESBL producers among the enterobacteriaceae was highest in Asia and the Middle East countries (>40%), while it was much less (<10%) in North America [23].

Our study showed a higher prevalence of resistance to most antibiotics tested. Among the tested antibiotics, only amikacin, piperacillin-tazobactam, vancomycin, and fosfomycin had acceptable sensitivity (<20%) (Table 3, 5). This prevalence pattern is similar to the comprehensive review by Mortazavi-Tabatabaei et al. [19] from Iran; Gajamer et al. [11]; and Shah et al. [6] from India (Table 5). Muhammad et al. [16] had recently reported a much higher prevalence of resistance for all antibiotics in Pakistan as compared to our study, with even carbapenem resistance reaching 40% (Table 5). The increased prevalence of carbapenem resistance in our study and similar geographical regions could be due to the higher prevalence of carbapenemase genes (97%) among the ESBL isolates, as reported by Gajamer et al., from India in 2019 [11]. On the other hand, Sokhn et al. from Lebanon had reported a much lesser prevalence of resistance for various antibiotics in their recent analysis (Table 5) [24]. In a study from the United States in 2017 across both community and hospital *E. coli *isolates, AMR prevalence was very low, with maximum resistance observed for cotrimoxazole (32%), while carbapenems had 100 percent sensitivity (Table 5) [25]. Our analysis shows that the prevalence of AMR is alarmingly high in the developing countries as compared to developed nations in concurrence with other authors [8,10]. 

**Table 5:** Prevalence of antimicrobial resistance reported across various studies and study settings (% prevalence) E. coli: *Escherichia coli*; Klebsiella sps: Klebsiella species All % approximated to the nearest whole numbers. Empty cells indicate that the testing and reporting for the particular antibiotic is not available from the study.

ANTIMICR OBIAL	Seyed Abdol Reza Mortazavi-Tabatabaei et al. [18] Iran - 2019		Varsharani Gajamer et al. [11] India - 2019	Ian A. Critchley et al. [25] USA - 2017	Vrushali Patwardhan et al. [26] India - 2017	Elie S. Sokhn et al. [24] Lebanon - 2020		Latika J Shah et al. [6] India - 2015		A. Muhammad et al. [17] Pakistan - 2020	Our study (2017 Data)
	E. coli	Klebsiella sps.	Enterobacteriaceae	E. coli	All uropathogens	E. coli	Kelbsiella sps.	E. coli	Kelbsiella sps.	All uropathogens	All uropathogens
Cotrimoxazole	54	64	75	32	63	45	39	83	71	86	60
Ampicillin	80	85	85	*	59			96	83		81
Amoxicillin	76	76									
Tetracycline	53	71									
Gentamicin	38	32	15	12	24	17	18	77	72	64	47
Amikacin	27	21		0.1	12	22	22	36	58	64	18
Norfloxacin			30		41	30	36	71	76	96	62
Ciprofloxacin	19	28	66	26		30	34	67	65	84	63
Levofloxacin				24	54						80
Nalidixic	33	43									
Ceftazidime	40	40	78	9		27	25			90	62
Ceftriaxone	40	35				35	28				
Cefotaxime	38	42			54					92	70
Cefixime	53	45									
Cephalexin	67	61									77
Cefepime				9		24	21			77	
Cefalotin	55	60									
Imipenem	13	14	12	0		0	0			38	32
Nitrofurantoin	42	18	70		39			75	65	81	30
Fosfomycin						4	3			58	0
Meropenem						0	0			49	38.3
Piperacillin tazobactam			22	1		14	21			71	18
Cefuroxime			66	16		54	44			86	68

Trends in the AMR across six years

Trends of changes in antimicrobial susceptibility is an important component of antimicrobial surveillance. In the present study, among the widely tested antibiotics (>200 samples), there had been a significant increase in resistance in 2017 for imipenem, amikacin, and nitrofurantoin, which was statistically significant (p<0.05) as compared to 2011 (Table 3, Figure 1, 2). Among the selectively tested antibiotics (<100 samples), meropenem and ertapenem showed a statistically significant (p<0.005) increase in resistance proportion (Table 3, Figure 1, 2).

Several authors have reported increasing trends in AMR from various regions by comparing two or more periods. Patwardhan et al. had earlier reported a significant increase in AMR in North India over a period of five years [26]. In a study from another part of South India by Prasada et al. over five years on *E. coli *infections, there was a significant increase in resistance for quinolones and piperacillin-tazobactam [27]. The study also showed a significant increase in the prevalence of ESBL producers over the study period (45% in 2013 to 60% in 2017).

Arana et al. from Spain did a 12-year analysis of both hospitalized and ambulatory patients with UTI and concluded that there had been a significant increase in MDR (multi-drug resistance) prevalence from 2007 to 2014 [28].

Stapleton et al. from Dublin had reported from their 2017 study on both hospital- and community-acquired UTIs that the resistance for most antimicrobials for *E. coli *isolates had increased over 10 years, except for nitrofurantoin and gentamycin [29]. Similarly, Caskurlu et al. from Turkey had recently reported a significant increase in resistance for many antibiotics, including fosfomycin, over a period of five years (2014-2018) [30].

The above discussion shows that there has been an increasing trend in resistance in general across the world, though the rates and patterns of increase varied widely. The trend of increase is at an alarmingly higher in middle- and low-income countries, with carbapenem resistance rates approaching nearly 35%. This calls for urgent strengthening of AMR surveillance and implementation of strong AMS programmes in these regions.

**Figure 3:**
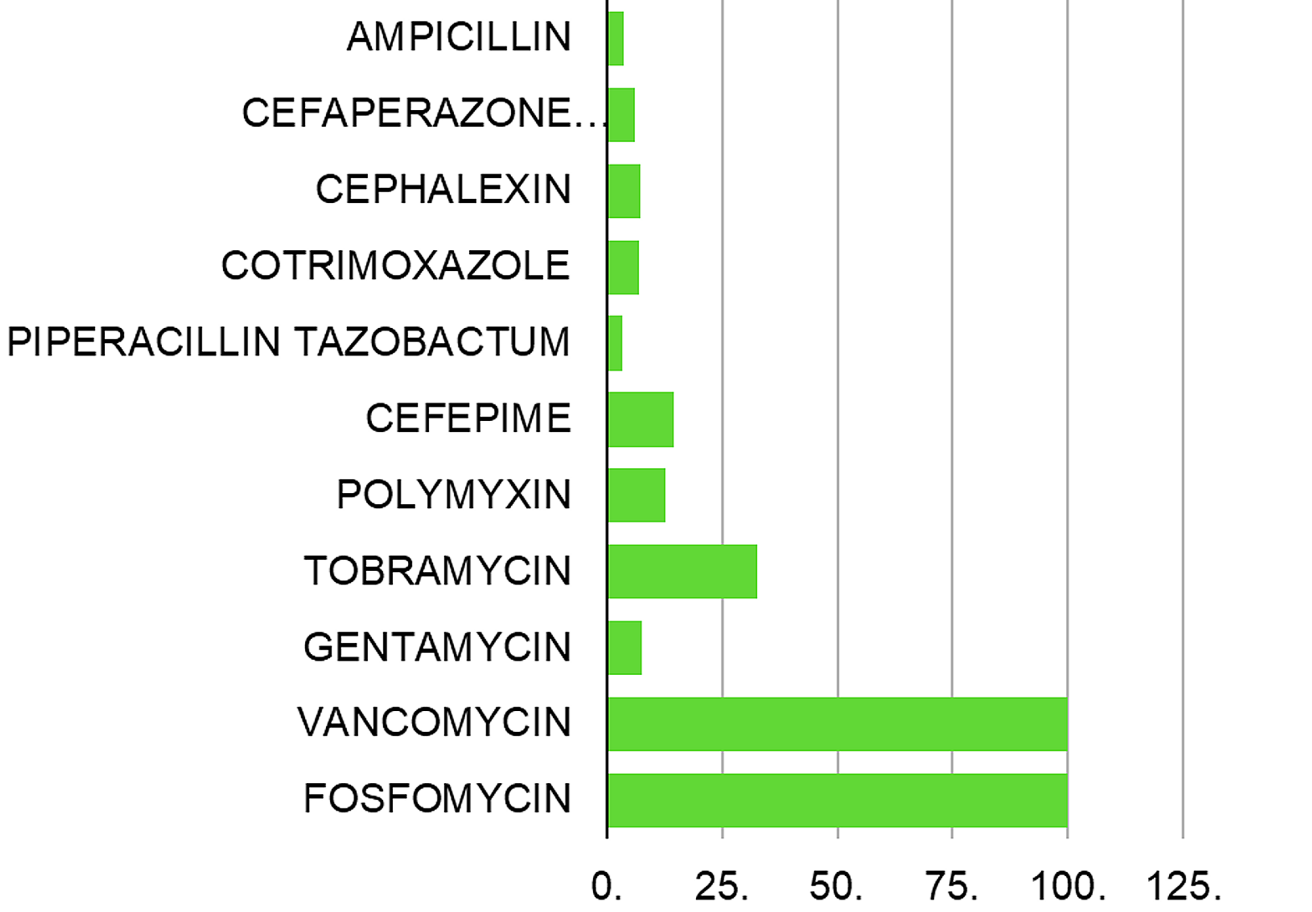
Increase in susceptibility (%) for antimicrobials observed in 2017 compared to 2011

On the positive side, our study showed increased susceptibility for many antibiotics, among which the increase for tobramycin and cephalexin in 2017 compared to 2011 was statistically significant (Table 3, Figure 3). However, there has been a lack of consistent reporting on the increasing susceptibility for antibiotics over the time period. Reporting of this might be useful in revising the guidelines for the treatment of UTI periodically.

Empirical choice of antibiotic for UTI

The choice of antibiotics for empirical treatment has been recommended to be based on the local AMR surveillance reports [1,6-9]. An AMR prevalence rate of more than 20% in a community for an antibiotic usually precludes its usage as an empirical choice [1,4]. In our study, only a few antibiotics had resistance rates lesser than 20% in 2017 (amikacin, piperacillin-tazobactam, linezolid, polymyxin, vancomycin, and fosfomycin) (Table 3, 5). Cefoperazone sulbactam, nitrofurantoin, and carbapenems had resistance rates between 20% to 40% (Table 3, 5). It is reasonable to conclude that fosfomycin is an excellent empirical choice for community-acquired UTI, considering the fact that the majority are caused by enterobacteriaceae, followed by nitrofurantoin, which can be used as an alternative. In the hospital setting, where resistant pathogens are suspected, amikacin or piperacillin-tazobactam might be the better choice for empirical treatment. Cefoperazone sulbactam can be used as a second-line agent. Usage of carbapenems for empirical treatment should be avoided and guided by the culture and sensitivity pattern. This might help in reducing the prevalence of resistance to this important reserve group of antibiotics.

Strengths of the study

This is the first study on the changing trends of prevalence in antimicrobial resistance over a period from this region. It might be a beginning point for more stringent surveillance protocol and AMS implementation in this region and other developing countries. For the first time, this study has correlated the influence of treatment guidelines and prevailing local AMR pattern on testing and reporting for various antibiotics. It has also highlighted the importance of reporting the increase in susceptibility for antibiotics, which might be useful in formulating region-specific treatment recommendations.

Limitations of the study

There are several limitations of our study. Cultures from paediatric population were not included in the study. Data on patient’s demography, risk factors, and clinical characters were not collected and analyzed. The data were not separately analyzed for hospitalized and non-hospitalized patients, which might influence the prevalence of resistance.

## Conclusions

Our analysis has shown that there is an alarmingly increasing trend for antimicrobial resistance (AMR) among uropathogens. It also shows a high prevalence of resistance for most antibiotics, including carbapenems, among the uropathogens in this part of India, similar to other developing nations. This warrants the implementation of stringent anti-microbial stewardship (AMS) programme and strengthening of the antimicrobial surveillance protocols in these regions. Data on changing trends of antimicrobial testing and reporting might help in strengthening antimicrobial surveillance. Based on the analysis, fosfomycin for community settings and either amikacin or piperacillin-tazobactam for hospital settings can be recommended as the empirical choice antimicrobials for urinary tract infection in this region.
